# CCR7-mediated T follicular helper cell differentiation is associated with the pathogenesis and immune microenvironment of spinal cord injury-induced immune deficiency syndrome

**DOI:** 10.3389/fnins.2022.1019406

**Published:** 2022-10-14

**Authors:** Chaochen Li, Chunshuai Wu, Guanhua Xu, Yang Liu, Jiajia Chen, Jinlong Zhang, Hongxiang Hong, Chunyan Ji, Zhiming Cui

**Affiliations:** ^1^The First People’s Hospital of Nantong, The Second Affiliated Hospital of Nantong University, Nantong University, Nantong, China; ^2^Key Laboratory for Restoration Mechanism and Clinical Translation of Spinal Cord Injury, Nantong, China; ^3^Research Institute for Spine and Spinal Cord Disease of Nantong University, Nantong, China

**Keywords:** spinal cord injury-induced immune deficiency syndrome, immune microenvironment, CCR7, biomarker, machine learning

## Abstract

Spinal cord injury-induced immune deficiency syndrome (SCI-IDS) is a disorder characterized by systemic immunosuppression secondary to SCI that dramatically increases the likelihood of infection and is difficult to treat. T follicular helper (Tfh) cells regulated by chemokine receptor CCR7 are associated with SCI-IDS after acute SCI. The present study explored the roles of CCR7 in SCI-IDS occurrence and immune microenvironment composition. Gene expression profile data of peripheral blood leukocytes from SCI and non-SCI subjects were collected from the Gene Expression Omnibus database. According to differential gene expression analysis, a protein-protein interaction (PPI) network, and risk model construction, the CCR7 expression level was prominently related to acute SCI and CCR7 expression was significantly downregulated after acute SCI. Next, we constructed a clinical prediction model and used it to identify patients with acute SCI. Using Gene Ontology (GO) analysis and gene set enrichment analysis (GSEA), we discovered that immune-related biological processes, such as T cell receptor signaling pathway, were suppressed, whereas chemokine-related signaling pathways were activated after acute SCI. Immune infiltration analysis performed using single sample GSEA and CIBERSORT suggested that Tfh cell function was significantly correlated with the CCR7 expression levels and was considerably reduced after acute SCI. Acute SCI was divided into two subtypes, and we integrated multiple classifiers to analyze and elucidate the immunomodulatory relationships in both subtypes jointly. The results suggested that CCR7 suppresses the immunodeficiency phenotype by activating the chemokine signaling pathway in Tfh cells. In conclusion, CCR7 exhibits potential as a diagnostic marker for acute SCI.

## Introduction

Spinal cord injury (SCI) is a common, serious injury associated with severe outcomes and high financial burden that causes SCI-induced immune deficiency syndrome (IDS) (Laginha et al., 2016; Wu et al., 2021). SCI-IDS can dramatically increase a patient’s susceptibility to pathogenic infections, including pneumonia, and delay wound healing, leading to increased morbidity, mortality, and complications (Sekhon and Fehlings, 2001; Riegger et al., 2007; Failli et al., 2012). Clinical research on death causes in SCI patients has emphasized the prevalence of pathogenic infections in these patients. In a 70-year study in the UK, the major cause of death in SCI patients was infections, such as pneumonia (23.5% of all deaths), with another 7.8% of deaths caused by urinary tract infections and unexplained sepsis (Savic et al., 2017). In the Czech Republic, the cause of death in SCI patients within the first year was peripheral infections (24.4%), and after 1 year, peripheral infections remained the main reason for their death, including pulmonary infections (14%), urinary tract infections (10.3%), sepsis of unknown origin (6.5%), and pressure sores (12.1%) (Kriz et al., 2021). Further, infection and associated hyperthermia can impair central nervous system (CNS) function after SCI (Inamasu et al., 2003; Meisel et al., 2004). Thus, clinically relevant infections are normal after SCI and significantly affect SCI prognosis.

Rapid and accurate diagnosis of SCI and mitigation of clinically pertinent infections of patients would ensure SCI patients’ recovery and their wellbeing. However, no quantitative diagnostic indicators for acute SCI are available. Acute SCI diagnosis is based on the 2011 International Standard for Neurological Classification of Spinal Cord Injury issued by the International Society for Spinal Cord Injury and the American Spinal Cord Injury Association (ASIA), ASIA Disability Classification, and comprehensive evaluation of conventional magnetic resonance images (Hulme et al., 2017; Rodrigues et al., 2018). The scoring criteria are based on the patient’s neurological damage and are used to determine patient prognosis based on clinical experience, which is subjective, and diagnosis is difficult as traumatic SCI frequently appears in multiple injuries (Galeiras Vazquez et al., 2017). Further, although SCI-IDS can be treated to improve the outcome of SCI by eliminating the major factors contributing to poor recovery, the precise immune mechanism is unknown and no therapeutic target is available (Meisel et al., 2005).

Current research suggests that the balanced interaction of the CNS with the immune system can be disturbed after SCI, which is an essential mechanism of escalation to SCI-IDS and infection (Meisel et al., 2005). Following SCI, supraspinal control of the sympathetic nervous system is damaged and sympathetic nerves are dysfunctional, leading to the rapid release of glucocorticoids from the adrenal glands, thus damaging immune function (Prüss et al., 2017). In patients with SCI above T6 thoracic level, sympathetic hyperreflexia is observed, which leads to an abnormal increase in norepinephrine in the spleen and the activation of beta-adrenergic receptors on lymphocytes, leading to consistent immunosuppression (Lucin et al., 2007; Zha et al., 2014).

Various treatment approaches for SCI-IDS, including androgen receptor therapy, glucocorticoids, and neuromodulation, have been tested, but have low clinical usability because of limited sensitivity and specificity (Shi et al., 2013; Koopman et al., 2016; Ueno et al., 2016; Pavlov and Tracey, 2017). A recent study revealed that the spleen can serve as a therapeutic target to restore immune homeostasis of the body after immunosuppression following high-segment SCI (Noble et al., 2018). Notably, incompletely SCI, such as moderate to severe spinal cord contusions, can dramatically alter the level-dependence of SCI-IDS (Hong et al., 2019). However, these previous studies did not focus on differential immune cell expression and did not explore the underlying molecular mechanisms. Further research is needed to explore the pathogenesis of SCI-IDS to improve its diagnosis and treatment.

In the recent field of immune microenvironment research, T follicular helper (Tfh) cells have garnered attention because of their important roles in immune system establishment and functional refinement. Peripheral immunosuppression and impaired Tfh cell function are closely associated in patients with severe CNS injury (Quattrocchi et al., 1991). Evidence indicates that Tfh cells have significant effects on the immunosuppressive process. For example, Tfh cells cause serious immune deficiencies due to aging (Almanan et al., 2020) and in patients with chronic inflammatory breast cancer, Tfh cells have been found to convert immunosuppression into an antitumor humoral response (Gu-Trantien et al., 2017). Tfh cells represent a particular CD4 + T cell subset of the lymph nodes and spleen and they contribute to information transfer in B cell differentiation, B cell activation, and germinal center formation (Crotty, 2014). The chemokine receptors CXCR5 and CCR7 allow the migration of Tfh cells to the T cell–B cell border for Tfh cell differentiation and maturation (Meli et al., 2016; Vinuesa et al., 2016). It has been demonstrated that that CCR7 is associated with the pathological processes of immunosuppressive diseases. For example, in myeloid cells, overexpression of CCR7 facilitated the targeted transfer of macrophages to the lymph nodes, thereby mediating immunosuppression (Yu et al., 2021).

This study aimed to identifying peripheral blood markers for accurate diagnosis of acute SCI and to interpreting changes in the immune microenvironment of SCI-IDS, using a high-throughput multi-omics approach. The potential association of CCR7 with SCI-IDS pathogenesis and changes in the immune microenvironment were examined using bioinformatics analyses, including differential gene expression analysis, protein-protein interaction (PPI) network analysis, scale-free network centrality analysis, clinical prediction model construction, functional enrichment analysis, molecular subtype analysis, and immune infiltration analysis combined with machine learning model analysis.

## Materials and methods

### Data sources and preprocessing

Chip-based RNA-sequencing data from peripheral blood leukocytes of acute SCI patients and controls (GSE151371) (Kyritsis et al., 2021) were collected from the Gene Expression Omnibus (GEO) database.^1^ The dataset comprises data from 10 healthy control subjects (HC group), 10 trauma patients without CNS injury (TC group), and 38 acute SCI patients (SCI group). The mRNA data were log_2_ transformed after filtering a minimum of 70% valid values. Quantile normalization of the data was carried out using the Bioconductor package limma. Clinical data from the SCI patients, including censored data such as the ASIA impairment scale, were acquired from Kyritsis et al. (2021) (Table 1).

**TABLE 1:** Baseline information.

Characteristic	SCI	HC	TC
n*	38	10	10
**Status, n (%)**			
non-SCI	0 (0%)	10 (17.2%)	10 (17.2%)
SCI	38 (65.5%)	0 (0%)	0 (0%)
**Gender, n (%)**			
Female	13 (22.4%)	2 (3.4%)	4 (6.9%)
Male	25 (43.1%)	8 (13.8%)	6 (10.3%)
**Race, n (%)**			
Asian	3 (5.2%)	0 (0%)	1 (1.7%)
Black or African-American	3 (5.2%)	2 (3.4%)	0 (0%)
Hispanic	17 (29.3%)	4 (6.9%)	4 (6.9%)
Other	0 (0%)	0 (0%)	3 (5.2%)
Unknown	10 (17.2%)	4 (6.9%)	2 (3.4%)
White	5 (8.6%)	0 (0%)	0 (0%)
**Prior CNS pathology, n (%)**			
No	21 (36.2%)	10 (17.2%)	10 (17.2%)
Unknown	5 (8.6%)	0 (0%)	0 (0%)
Yes	12 (20.7%)	0 (0%)	0 (0%)
**Traumatic brain injury, n (%)**			
No	28 (48.3%)	10 (17.2%)	10 (17.2%)
Unknown	2 (3.4%)	0 (0%)	0 (0%)
Yes	8 (13.8%)	0 (0%)	0 (0%)
**Neurological level, n (%)**			
Cervical	18 (31%)	0 (0%)	0 (0%)
Lumbar	2 (3.4%)	0 (0%)	0 (0%)
Thoracic	10 (17.2%)	0 (0%)	0 (0%)
Unknown	8 (13.8%)	10 (17.2%)	10 (17.2%)
**Age (mean ± SD)**	55.26 ± 20	49.44 ± 12.2	41.7 ± 17.98
Time of injury (median (IQR)**)	23 (17, 43)		20 (19.25, 22.25)

*n, number of samples. **IQR, interquartile range.

### Differential gene expression analysis

Differential gene expression among the HC, TC, and SCI groups was analyzed using the R package limma (Phipson et al., 2016) based on cutoffs of |log fold change| ≥ 1.5 and adjusted *p* < 0.05. In addition, differential expression between pairs of groups (HC vs. TC, HC vs. SCI, and TC vs. SCI) was analyzed. Only those genes with significant and specific changes in expression after SCI were selected (*n* = 555).

### Protein-protein interaction network construction

A PPI network of the differentially expressed genes was constructed using the STRING 11.5 database (Szklarczyk et al., 2019), with a confidence level of 0.7. To identifying highly connected subnetworks, the MCODE clustering algorithm with default parameters was applied to the network in Cytoscape (version:3.9.1) (Shannon et al., 2003). Genes in the top 2 clusters were considered as hub genes for downstream analysis. In a PPI network, node strength indicates the importance of a node according to the strength of its connections to the other network nodes. Degree centrality, betweenness centrality, closeness centrality, and stress centrality of nodes were selected as metrics of node centrality, and we calculated the degree of node centrality for each node in the PPI network using Cytoscape. Then, the intersection of the top 5 results of the four centrality analyses was taken.

### Construction of risk models and nomogram prediction models

We created risk models to analyze the correlation between differentially expressed genes and acute SCI. First, we determined key genes related to acute SCI through univariate LR analysis. Second, the least absolute shrinkage and selection operator regression was applied in dimensionality reduction analysis to verify key genes related to acute SCI. Then, odds ratios, 95% confidence intervals and *p*-values were determined using univariate and multivariate LR. We selected only significant variables (*p* < 0.05) after multivariate LR analysis to develop nomogram prediction models for acute SCI. Model performance was evaluated using receiver operating characteristic curve (ROC) and calibration curves, representing the probability that the classifier will correctly label a new patient.

### Gene set enrichment analysis (GSEA) and functional enrichment analysis

GSEA is a computational approach to analyze if any specific gene set shows statistically significant differences between two biological states and frequently applied in estimating changes in pathways and biological process activity in expression dataset samples (Subramanian et al., 2005). Differences in biological processes between groups were investigated by GSEA using the clusterProfiler package (Yu et al., 2012) and the GSE151371 dataset. Gene Ontology (GO) term enrichment and Kyoto Encyclopedia of Genes and Genomes (KEGG) pathway enrichment analyses of the 555 differentially expressed genes were conducted using the clusterProfiler package.

### Immune infiltration analysis

Immune cell infiltration levels in the HC, TC, and SCI groups were estimated using single-sample (ss) GSEA in the GSVA (Hanzelmann et al., 2013) R package. ssGSEA determines the immune cell population in a sample base on gene expression data (Subramanian et al., 2005). CIBERSORT, an analytical tool developed by Newman et al. (2019) that estimates cell type abundance in a mixed cell population based on gene expression data, was used to validate immune infiltration results.

### Analysis of molecular subtypes and their immune microenvironments

To identify the acute SCI subtypes, univariable LR was applied to identify genes related to ASIA levels in the SCI group, with a cutoff of *p* < 0.05. Then, the R package ConsensusClusterPlus (Wilkerson and Hayes, 2010) (reps = 1,000 bootstraps, pItem = 0.8, pFeature = 0.8) was used for class discovery. An average pairwise consensus matrix of consensus clusters, a delta plot of the relative change of the area under the consensus cumulative distribution function (CDF) curve, and the average silhouette distance of the consensus clusters were used to determine the number of clusters. Principal coordinate analysis was used to validate the molecular subtype analysis results. We selected two acute SCI clusters based on consensus clustering. To analyze the immune microenvironment in the two consensus molecular subtypes, we profiled the immune infiltration analysis in each subtype.

### Immune infiltration feature identification

Patients with acute SCI were classified according to the ASIA impairment scale, with grades A, B, and C forming the ASIA-high group and grades D and E forming the ASIA-low group. Appropriate immune infiltrating cells in each subtype related to ASIA levels were selected based on ensemble models, such as extremely logistic regression (LR), a gradient boosting decision tree (GBDT), a random forest (RF), or extreme gradient boosting (XGBoost). Infiltrating immune cell importance scores were computed based on these models. Next, we obtained the immune infiltrating cells (|log fold change| ≥ 0.25 and false discovery rate < 0.05) between the ASIA-high and ASIA-low groups in the two molecular subtypes using the Wilcoxon test. The intersection of results of the five methods (LR, GBDT, RF, XGBoost, and the Wilcoxon test) was taken as the final immune infiltrating cells associated with ASIA levels.

### Correlation analysis between immune microenvironment and acute spinal cord injury

To analyze the correlations between the immune microenvironment and acute SCI, first, correlations between a potential biomarkers (CCR7) and two acute SCI molecular subtypes (Cluster1, Cluster2) were determined using Spearman’s correlation analysis. Next, Spearman correlation analysis between the potential biomarker and final immune infiltrating cells associated with ASIA levels was conducted.

### Statistical analysis

The R software (version 4.2.0) was used for data processing and analyses. For comparing two groups of continuous variables, the independent Student’s *t*-test was used for normally distributed data and the Mann-Whitney *U*-test (Wilcoxon rank-sum test) for non-normally distributed data. The chi-square test or Fisher’s exact test was used to compare two groups of categorical variables. Correlation coefficients of differentially expressed genes were determined using Spearman correlation analysis. All *p*-values were two-sided, and *p* < 0.05 was considered statistically significant.

## Results

### Differential gene expression analysis

**FIGURE 1:**
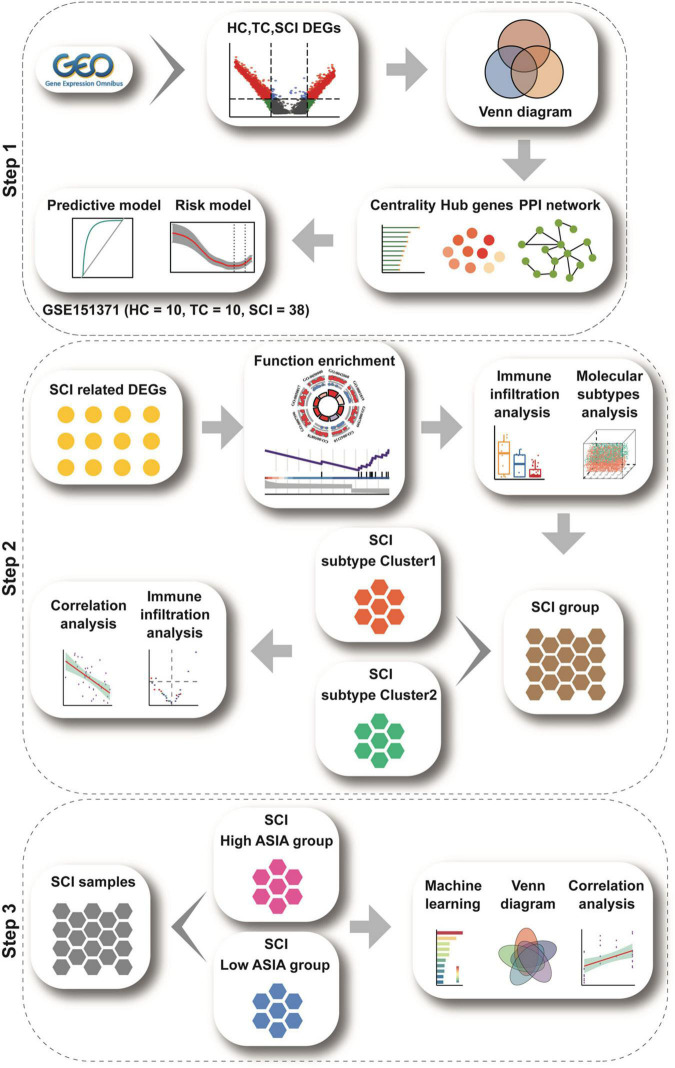
Study flow chart. The study flow chart is divided into three main sections according to the study sequence. Each icon represents schematically an analysis or a collection of data to be analyzed.

A flow chart of the study is presented in Figure 1.

**FIGURE 2:**
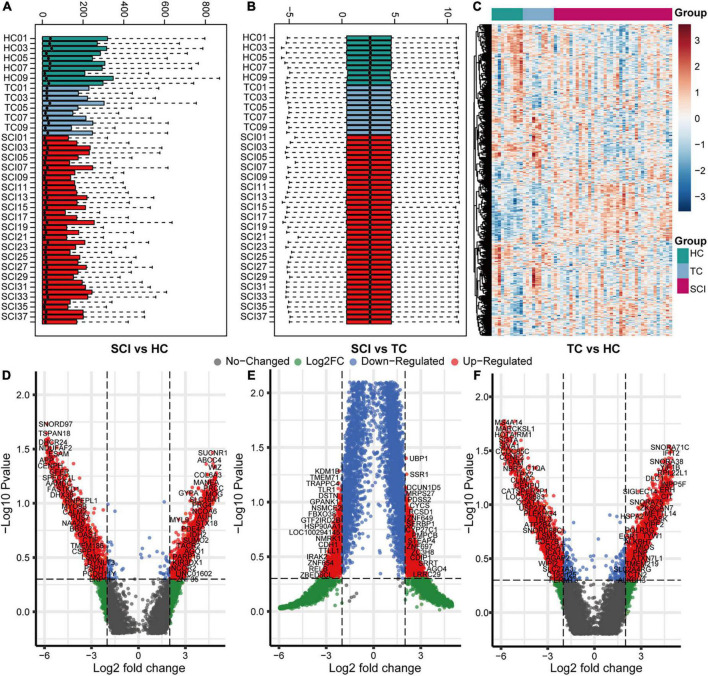
Overall gene expression profiles of peripheral blood leukocytes in acute SCI patients. **(A,B)** GSE151371 chip data before and after background correction. **(C)** Heatmap of the overall expression of GSE151371 chip data. **(D–F)** Volcano plots of differentially expressed genes between the SCI and HC groups, SCI and TC groups, and TC and HC groups.

To analyze the overall gene expression profile of patients with acute SCI, we comprehensively analyzed RNA-sequencing data from peripheral blood leukocytes of subjects in the HC, TC, and SCI groups in the GSE151371 dataset, using background correction (Figures 2A–C). To evaluate the molecular mechanisms of the changes induced by acute SCI, differentially expressed genes among the three groups were identified (Figures 2D–F). We found 555 genes that were significantly altered specifically in the SCI population based on cutoffs of |log fold change| ≥ 1.5 (Supplementary Table 1) and a false discovery rate and adjusted *p* ≤ 0.05 (Supplementary Figure 1).

### Protein-protein interaction network analysis

**FIGURE 3:**
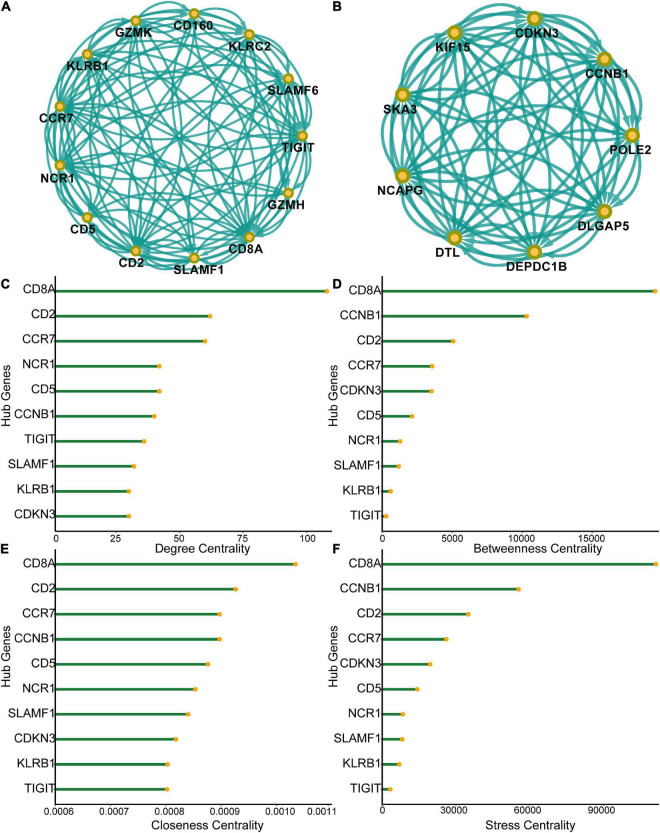
Protein-protein interaction (PPI) network of differentially expressed genes. **(A)** Subnetworks of the top-ranked PPI network. **(B)** Subnetworks of the second-ranked PPI network. **(C–F)** Analysis of degree centrality, betweenness centrality, closeness centrality and stress centrality in the top two PPI network subnetworks.

A PPI network of the 555 differentially expressed genes was constructed based on the STRING database to identify differences between the SCI and control groups. Four hundred and seventy-seven genes were mapped to the network, of which 477 genes were interconnected with an average local clustering coefficient of 4.65. From the PPI network, 15 MCODE modules were identified (see Supplementary Appendix for details). To quantify the importance of the differentially expressed genes in the PPI network, the two most important MCODE components were extracted for further analysis (22 hub genes) (Figures 3A,B). The top 10 genes in the PPI network in terms of degree centrality, betweenness centrality, proximity centrality, and stress centrality are shown in Figures 3C–F. Three of the 22 hub genes, *CD8A*, *CD2*, and *CCR7*, were found in the top 5 results of the four centrality analyses, suggesting that they may be crucial in acute SCI.

### Construction of risk models and nomogram prediction models

**FIGURE 4:**
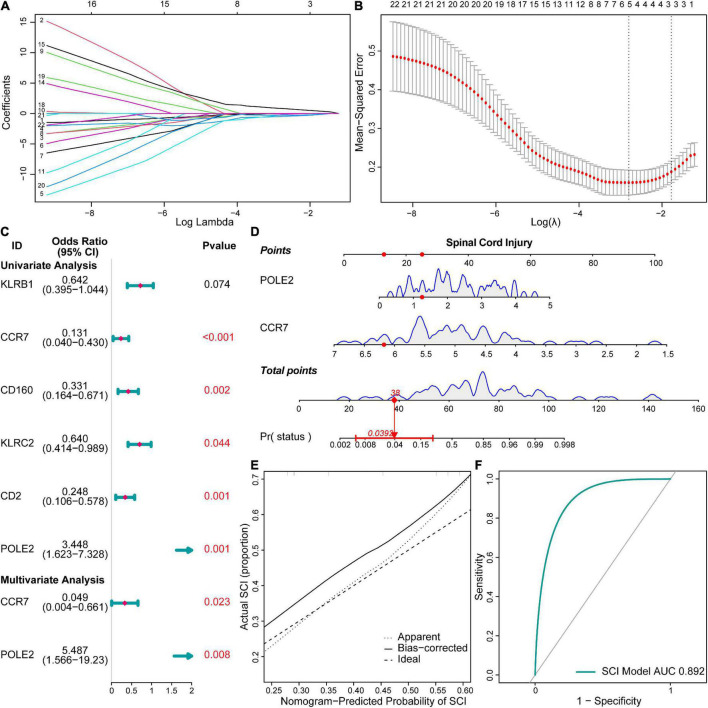
Risk models and clinical prediction models for acute SCI. **(A,B)** Least absolute shrinkage and selection operator regression identified key genes in acute SCI. **(C)** Forest plot for logistic regression. **(D)** Nomogram prediction model. **(E)** Calibration curve for the nomogram prediction model. **(F)** Receiver operating characteristic (ROC) curve of the nomogram prediction model.

We constructed a risk model to determine the risk factors of acute SCI. We found that CCR7 and POLE2 were independent risk factors for the development of acute SCI (Figures 4A–C). A nomogram plot containing these two predictors was constructed, with an area under the ROC curve of 0.892 (Figures 4D,F). Calibration curves corroborated the good performance of the nomogram (Figure 4E). To evaluate the clinical application potential of the prediction model, we randomly selected a sample from a healthy subject for prediction, and the prediction results suggested that the probability of acute SCI onset in this subject was 3.92% (Figure 4D). Although multivariate LR suggested that *POLE2* and *CCR7* are independent risk factors for acute SCI, *POLE2* does not play a key role in acute SCI according to the PPI network analysis. Based on all these results, we finally selected *CCR7* for further study.

### Gene set enrichment analysis and functional enrichment analysis

**FIGURE 5:**
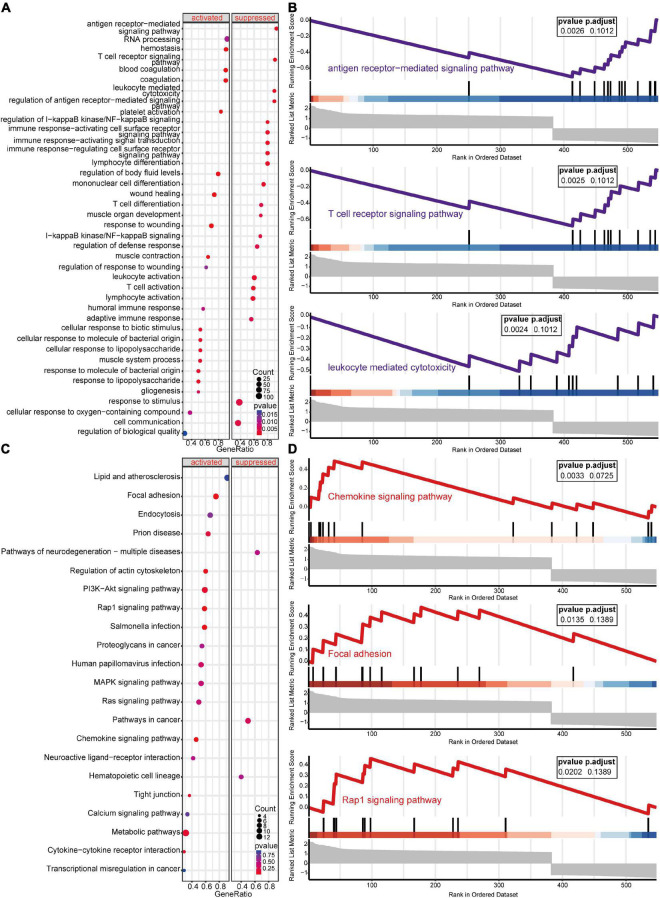
Gene set enrichment analysis (GSEA) of the experimental and control groups. **(A)** Bubble plot of GSEA-Gene Ontology (GO) analysis results. The horizontal coordinate indicates the gene ratio, the vertical coordinate the GO terms, dot size indicates gene number, dot color the *p*-value, left and right separation indicate activation and repression. **(B)** Clustering of the first three items of GSEA-GO terms. **(C)** Bubble plot of GSEA-KEGG results. The horizontal coordinate indicates the gene ratio, the vertical coordinate the GO terms, dot size indicates gene number, dot color the *p*-value, left and right separation indicate activation and repression. **(D)** GSEA-KEGG analysis of the first three clusters.

GSEA-GO and GSEA-KEGG are enrichment methods to identify classes of genes or proteins that are over-represented in a large set of genes or proteins. The top 5 terms identified in GSEA are listed in Table 2. GSEA-GO enrichment results revealed that antigen receptor-mediated signaling pathway regulation, T cell receptor signaling pathway, and leukocyte mediated cytotoxicity, were suppressed after acute SCI (*p* < 0.01) (Figures 5A,B). GSEA-KEGG enrichment results revealed that the chemokine signaling pathway and other chemokine-related pathways were activated after acute SCI (*p* < 0.01) (Figures 5C,D).

**TABLE 2:** Top five terms in GSEA.

Category	ID	Enrichment score	Normalized enrichment score	*P*
BP	GO:0009611	4.65E-01	2.99E + 00	1.49E-03
BP	GO:0042060	5.06E-01	3.10E + 00	1.53E-03
BP	GO:0007596	6.48E-01	3.23E + 00	1.55E-03
BP	GO:0050817	6.48E-01	3.23E + 00	1.55E-03
BP	GO:0050878	4.86E-01	2.78E + 00	1.56E-03
MF	GO:0005509	4.20E-01	2.30E + 00	1.56E-03
MF	GO:0030545	4.55E-01	1.98E + 00	3.18E-03
MF	GO:0030546	4.55E-01	1.98E + 00	3.18E-03
MF	GO:0048018	4.55E-01	1.98E + 00	3.18E-03
MF	GO:0038023	−2.44E-01	−2.19E + 00	3.31E-03
CC	GO:0005615	3.11E-01	2.84E + 00	1.30E-03
CC	GO:0099503	4.00E-01	2.90E + 00	1.45E-03
CC	GO:0030141	4.43E-01	3.13E + 00	1.46E-03
CC	GO:0031091	6.40E-01	2.92E + 00	1.58E-03
CC	GO:0031983	4.55E-01	2.39E + 00	1.59E-03
KEGG	hsa04062	4.90E-01	1.99E + 00	3.29E-03
KEGG	hsa04510	4.73E-01	1.86E + 00	1.35E-02
KEGG	hsa04015	4.59E-01	1.81E + 00	2.02E-02
KEGG	hsa05132	4.40E-01	1.73E + 00	2.53E-02
KEGG	hsa04810	4.54E-01	1.65E + 00	4.16E-02

Unlike GSEA-GO and GSEA-KEGG, original GO and KEGG enrichment analyses are based on hypergeometric distributions. To accurately obtain functional changes after SCI, GOs and KEGG enrichment analyses were performed to test the stability of the GSEA enrichment results (Table 3). The results indicated that the top 5 functions related to immunity in GO biological processes, namely positive regulation of cytokine production, T cell activation, leukocyte migration, antigen receptor-mediated signaling pathway regulation, and leukocyte chemotaxis, were significantly enriched (Supplementary Figures 2A–D). The 555 differentially expressed genes were mapped to the KEGG pathways, and the top 15 enriched KEGG pathways are shown in Supplementary Figures 2E,F. Taken together, the data suggested that peripheral immune function was suppressed and chemokine-related signaling pathways were activated after acute SCI.

**TABLE 3:** Top five terms in GO and KEGG enrichment analyses.

Category	ID	Description	*P*-value
BP	GO:0042060	Wound healing	6.97E-09
BP	GO:0001819	Positive regulation of cytokine production	2.62E-08
BP	GO:0007599	Hemostasis	9.72E-08
BP	GO:0042110	T cell activation	2.27E-07
BP	GO:0050878	Regulation of body fluid levels	2.73E-07
MF	GO:0140375	Immune receptor activity	6.31E-05
MF	GO:0023023	Major histocompatibility complex binding	2.34E-04
MF	GO:0005516	Calmodulin binding	5.16E-04
MF	GO:0001618	Virus receptor activity	5.84E-04
MF	GO:0140272	Exogenous protein binding	6.38E-04
CC	GO:0031091	Platelet alpha granule	1.49E-10
CC	GO:0031093	Platelet alpha granule lumen	1.36E-08
CC	GO:0034774	Secretory granule lumen	3.61E-06
CC	GO:0060205	Cytoplasmic vesicle lumen	4.24E-06
CC	GO:0031983	Vesicle lumen	4.71E-06
KEGG	hsa04610	Complement and coagulation cascades	9.21E-04
KEGG	hsa05202	Transcriptional misregulation in cancer	1.77E-03
KEGG	hsa04612	Antigen processing and presentation	2.16E-03
KEGG	hsa05414	Dilated cardiomyopathy	2.18E-03
KEGG	hsa04062	Chemokine signaling pathway	4.83E-03

### Immune infiltration analysis between SCI and control groups

**FIGURE 6:**
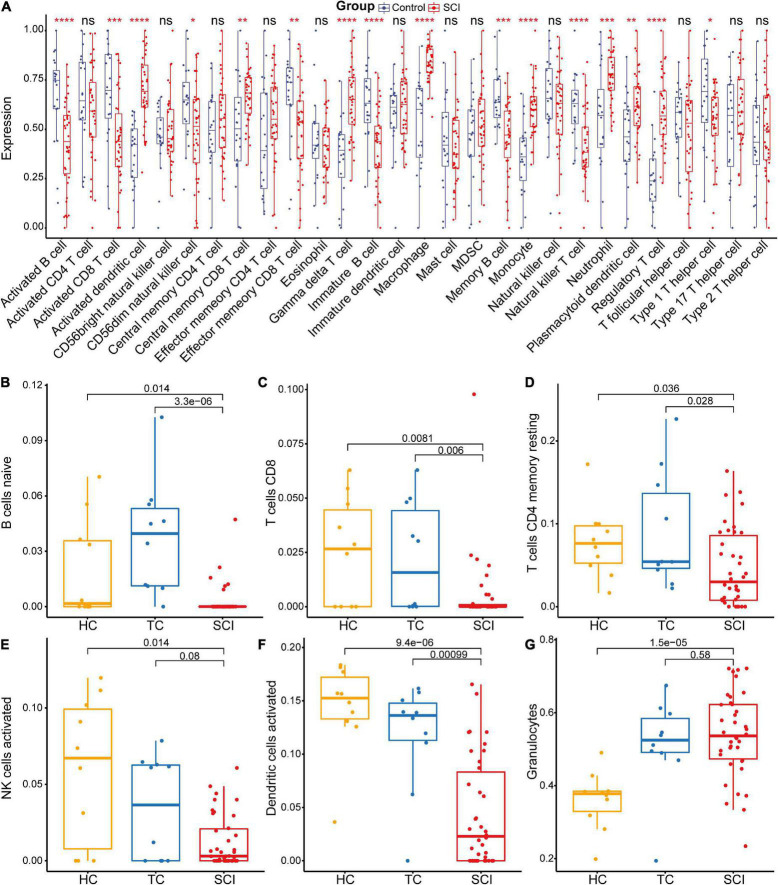
Acute SCI immune infiltration analysis. **(A)** Differential immune cell infiltration in the experimental and control groups based on ssGSEA. “ns” indicates *p* ≥ 0.05, *p < 0.05, **p < 0.01, ***p < 0.001, and ****p < 0.0001. **(B–G)** Differential immune cell infiltration in the experimental and control groups according to CIBERSORT analysis. The number above the box indicates the *p*-value.

The gene functional annotation results suggested that peripheral immune function was suppressed after acute SCI. To explore the mechanism of SCI-IDS, immune cell infiltration abundance was measured using ssGSEA (Figure 6A). The results indicated that the levels of some immune infiltrating cells, including activated CD8 T cells, activated B cells, and CD56dim natural killer (NK) cells, were significantly decreased in the SCI group compared to the control group (non-SCI patients) (*p* < 0.05). The CIBERSORT algorithm was used to determine the abundance of peripheral immune infiltrating cells after acute SCI (Figures 6B–G). The results showed that naive B cells naive, CD8 T cells, CD4 memory resting T cells, activated NK cells, and activated dendritic cells contents were significantly decreased after acute SCI. The immune infiltration analysis results provided evidence for the development of SCI-IDS after acute SCI.

### Establishment of molecular subtypes of acute spinal cord injury

**FIGURE 7:**
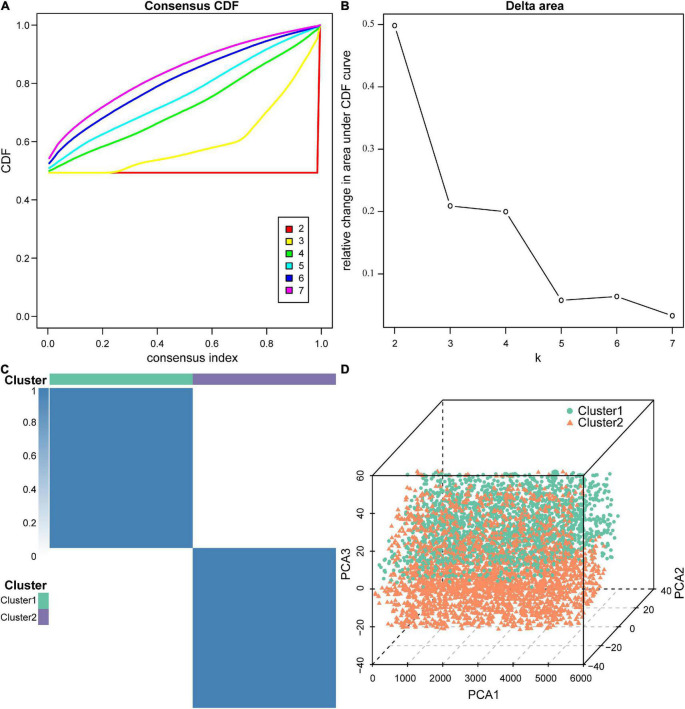
Molecular subtypes of acute SCI. **(A)** Cumulative distribution function (CDF) curve of consensus clustering of acute SCI-related genes. The horizontal coordinate indicates the consensus index, the vertical coordinate indicates the CDF index. **(B)** Relative change in the area under the CDF curve revealing two subtypes with the most stable trend of change. **(C)** Clustering heatmap of the subtypes of acute SCI-related genes. **(D)** Principal coordinate analysis plot for acute SCI-related molecular subtypes.

Next, we performed molecular subtype analysis using unsupervised clustering to obtain a more accurate biomarker. First, based on ASIA scores, a univariate LR analysis of the differentially expressed genes in the 38 acute SCI patients was conducted, and genes that were highly correlated with ASIA scores were screened out (*p* < 0.05) (see Supplementary Appendix for details). Next, we constructed the molecular subtypes of acute SCI based on the univariate LR results. CDF curves suggested that the optimal clustering of acute SCI was into two subtypes: Cluster 1 and Cluster 2 (Figures 7A–C). Principal coordinate analysis was used to validate the molecular subtype analysis results; the results indicated that Cluster 1 and Cluster 2 were well separated (Figure 7D).

### Immune infiltration feature identification

**FIGURE 8:**
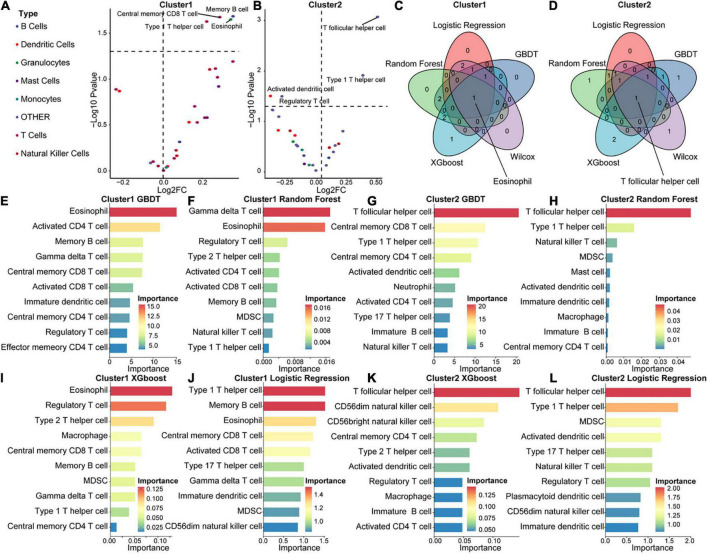
Immunological composition and feature identification analysis of the molecular subtypes of acute SCI. **(A,B)** Volcano plots of differential expression of immune infiltrating cells in ASIA-high and -low groups in acute SCI subtypes Cluster 1 and Cluster 2. **(C,D)** Venn diagrams of logistic regression (LR), random forest (RF), GBDT, XGboost, and Wilcoxon analyses in the acute SCI subtypes Cluster 1 and Cluster 2. **(E,F)** Histogram of GBDT and RF classifiers in the acute SCI subtype Cluster 1. The horizontal coordinates and colors indicate importance and the vertical coordinate indicates immune infiltrating cells. **(G,H)** Histogram of GBDT and RF classifiers in acute SCI subtype Cluster 2. The horizontal coordinates and colors indicate importance and the vertical coordinate indicates immune infiltrating cells. **(I,J)** Histogram of XGboost and LR classifiers in the acute SCI subtype Cluster 1. The horizontal coordinates and colors indicate importance and the vertical coordinate indicates immune infiltrating cells. **(K,L)** Histogram of XGboost and LR classifiers in the acute SCI subtype Cluster 2. The horizontal coordinates and colors indicate importance and the vertical coordinate indicates immune infiltrating cells.

To explore differences in the immune microenvironment between the two molecular acute SCI subtypes, we performed immune infiltration analysis of the two molecular subtypes separately. Base on the ASIA score, patients were categorized into two groups (ASIA-high and ASIA-low groups), followed by analysis of the differential immune cells infiltration in both groups using the Wilcoxon test, with a cutoff of false discovery rate < 0.05 (Figures 8A,B). The results suggested that memory B cells, CD8 central memory T cells, eosinophils, and type1 T helper cells were remarkably decreased in the ASIA-high group of the Cluster 1 subtype. In the Cluster 2 subtype, Tfh cells and type 1 T helper cells were significantly decreased, whereas regulatory T cells and activated dendritic cells were increased in the ASIA-high group.

In addition, correlations between immune infiltrating cells and ASIA scores in the two molecular subtypes were analyzed using four machine learning models, including LR, RF, GBDT, and XGBoost, for each subtype to obtain more accurate and stable results. These machine learning models are all classifier architectures and have good interpretability. We selected the top 5 immune infiltrating cells identified from the four machine learning models and took the intersection with the differentially expressed immune infiltrating cells (Figures 8E–L). Based on the combined results, we found that eosinophils were the key immune infiltrating cells that affected the ASIA score in subtype Cluster 1, and Tfh cells were the key immune infiltrating cells that affected the ASIA score in subtype Cluster 2 (Figures 8C,D).

### Correlation analysis between the immune microenvironment and acute spinal cord injury

**FIGURE 9:**
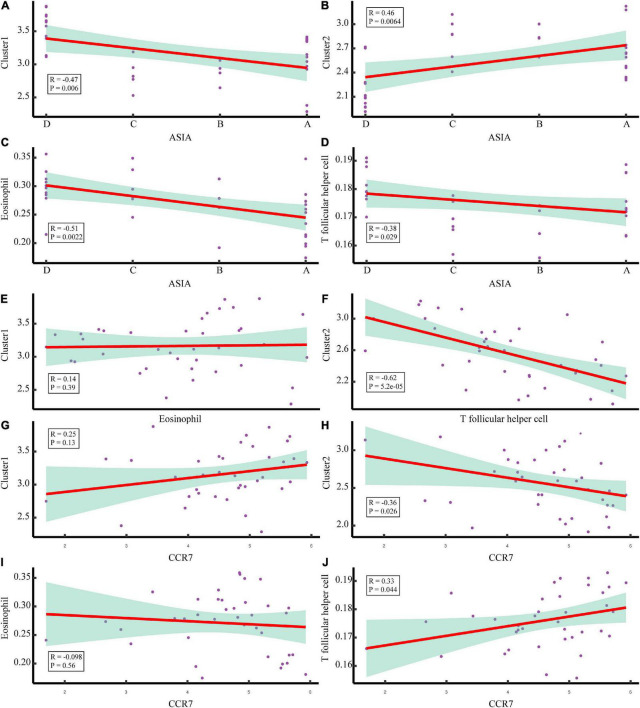
Correlation analysis of the molecular subtypes of acute SCI. **(A,B)** Scatter plot of correlation analysis between ASIA classification and the acute SCI molecular subtypes Cluster 1 and Cluster 2. **(C,D)** Scatter plot of correlation analysis between ASIA grading, eosinophils, and T follicular helper (Tfh) cells. **(E)** Correlation analysis of the acute SCI molecular subtype Cluster 1 and eosinophils. **(F)** Correlation analysis of the acute SCI molecular subtype Cluster 2 and Tfh cells. **(G,H)** Scatter plot of correlation analysis between CCR7 and the acute SCI molecular subtypes Cluster 1 and Cluster 2. **(I,J)** Scatter plot of correlation analysis between CCR7, eosinophils, and Tfh cells. R denotes the correlation coefficient, and *P* denotes the *p*-value.

To explore the immune microenvironment regulatory network of the two acute SCI subtypes, we conducted a series of Spearman correlation analyses. The Spearman test was used because the data did not follow a strictly normal distribution. First, we investigated the correlation of chemokine receptor CCR7 with acute SCI molecular subtypes and key immune infiltrating cells in both molecular subtypes. The results suggested that CCR7 was not significantly correlated with molecular subtypes Cluster 1 and eosinophils (*p* > 0.05), negatively correlated with molecular subtype Cluster 2 (*R* = –0.36, *p* = 0.044), and positively correlated with Tfh cells (*R* = 0.33, *p* = 0.044) (Figures 9G–J). Second, the association of critical immune infiltrating cells with acute SCI molecular subtypes and ASIA scores was studied. There were no significant relationships between eosinophils and subtype Cluster 1 and ASIA scores (*p* > 0.05), and Cluster 2 and ASIA scores were positively correlated (*R* = 0.46, *p* = 0.0064), whereas Tfh cells and ASIA scores were negatively correlated (*R* = –0.38, *p* = 0.029) (Figures 9C–F). In addition, we explored the correlation between the subtypes and ASIA scores, and the results suggested that subtype Cluster 1 and ASIA scores were negatively correlated (*R* = –0.47, *p* = 0.006), whereas subtype Cluster 2 and ASIA scores were positively correlated (*R* = 0.46, *p* = 0.0064) (Figures 9A,B). Notably, CCR7 was negatively correlated with ASIA scores, and was significantly lowly expressed in the ASIA-high group (Supplementary Figures 3A,B). This suggested that the downregulation of CCR7 and suppression of Tfh cells after acute SCI cause an increase in the ASIA score.

## Discussion

SCI is considered a common and severe illness with an increasing rate of disability and mortality, often with catastrophic consequences (Byra, 2016; Casper et al., 2018). The significant effects of immunosuppression on Tfh cells have attracted much attention (Yan et al., 2017). Using differential gene analysis, PPI network centrality analysis, and risk model construction, we identified CCR7 as a possible peripheral blood diagnostic markers for acute SCI and a therapeutic target for SCI-IDS. Next, we constructed predictive models by comparing the SCI and control groups (non-SCI subjects). We discovered that the biological processes and pathways related to the immune response were the most enriched in acute SCI. The immune infiltration results suggested that SCI-IDS occurred immediately after acute SCI. Next, we established two molecular subtypes if acute SCI and identified risk factors using supervised machine learning models. Unlike in acute SCI subtype Cluster 1, Tfh cells positively regulated by CCR7 in molecular subtype Cluster 2 significantly positively affected acute SCI prognosis.

Numerous studies have suggested the associations of various SCI-IDS symptoms with the functions of macrophages, NK cells, T cells and B cells (Cruse et al., 1992; Campagnolo et al., 1997; Furlan et al., 2006; Lucin et al., 2007; Riegger et al., 2009; Held et al., 2010). Therefore, sequencing data of human peripheral blood leukocytes were used in the present study. We normalized the GSE151371 chip data to eliminate data bias. Differential gene analysis revealed that 555 genes were significantly altered specifically in acute SCI. *CCR7* expression was downregulated after acute SCI and was one of the potential biomarkers based on differential expression analysis. As a chemokine receptor, CCR7 exerts remarkable effects on the differentiation of Tfh cells, the homing of lymphocytes, and the formation of germinal centers (Forster et al., 1999; Hardtke et al., 2005; Arnold et al., 2007). CCR7 expression is significantly increased in patients with pancreatic cancer and induces intra-tumor angiogenesis and lymphangiogenesis through chemotactic interactions with its ligand, CCL21 (Zhao et al., 2011). CCR7 significantly affects multiple sclerosis relapse by regulating Tfh cell differentiation (Fan et al., 2015). Our research revealed the significant role of CCR7 in SCI-IDS.

From the PPI networks constructed in the current study, using sub-network extraction and centrality analysis, we identified *CD8A*, *CD2*, and *CCR7* as genes involved in acute SCI. The PPI analysis corroborated the importance of CCR7 in acute SCI from the scale-free network perspective. Both univariate and multivariate LRs suggested that CCR7 and POLE2 were significantly associated with acute SCI; however, only CCR7 showed high significance in both the scale-free network and linear regression, and was therefore selected as a potential biomarker for acute SCI. Its value in acute SCI and SCI-IDS diagnosis and treatment was further analyzed by constructing a clinical prediction model. GSEA-KEGG and KEGG functional enrichment analysis indicated that chemokine signaling and the PI3K/AKT pathway were significantly upregulated after acute SCI, suggesting an active chemokine cascade response. GSEA-GO and GO enrichment analysis indicated that chemokines became more active after acute SCI, while immune function was suppressed. Interestingly, a recent study showed that that chemokines play an important role in bone marrow failure caused by SCI (Carpenter et al., 2020). These results suggest that chemokines may play a role in the immunosuppressive process after acute SCI.

Regarding immune infiltration, Tfh cell function was damaged in SCI patients compared to control patients, mainly in terms of a decreases in the proportions of mature B cells and NK cells. This finding is consistent with previously reported experimental results (Kliesch et al., 1996). Further, we found that activated B cells were significantly reduced after acute SCI. It is well known that B cell maturation requires Tfh cell assistance to generate germinal centers, and CCR7-mediated effector cell migration also plays a key role in the formation of germinal centers (Salem et al., 2021). The downregulation of CCR7 after acute SCI may contribute to the impaired B cell activation. This was corroborated by the positive correlation between CCR7 expression and Tfh cell abundance. These evidences demonstrate the biomarker potential of CCR7 in SCI-IDS. Li et al. (2019) classified hepatocellular carcinoma into five immune subtypes, which can be targeted for treatment. Wang et al. (2021) classified SCI patients based on gene set variation analysis enrichment scores and found that Cluster 1 had the highest activity in the MAPK, NOTCH, MTOR, and WNT pathways. Similarly, we established two acute SCI subtypes and measured the correlation between ASIA scores and the two subtypes. Immunological infiltration was analyzed separately for the two subtypes. The results suggested that the two subtypes have different immunological characteristics. To identify the immune cells that exacerbate acute SCI disease, we built machine learning clusters in each of two molecular subtypes. Eosinophils and Tfh cells were found to play a major role in molecular subtype Cluster 1 and Cluster 2, respectively. There were no significant correlations between CCR7, Cluster 1, and eosinophils. This suggests that CCR7 is less suitable as a target for SCI-IDS of the molecular subtype Cluster 1. We found a reduced abundance of Tfh cells in the ASIA-high group of the molecular subtype Cluster 2, suggesting an effect of Tfh cells on neurological function in patients with acute SCI. Our results support that SCI-IDS aggravates acute SCI (Brommer et al., 2016). Moreover, CCR7 and the molecular subtype Cluster 2 were negatively correlated, whereas CCR7 and Tfh cells were positively correlated, suggesting that CCR7 plays an important role in the molecular subtype Cluster 2 and that downregulation of CCR7 after acute SCI causes a decrease in Tfh cell abundance. Tfh cells were negatively correlated with ASIA classification. This suggested that a decrease in Tfh abundance leads to an increase in the ASIA score. Combined with the functional enrichment findings that after acute SCI, peripheral immune function is suppressed and chemokine signaling pathways are activated, we suggest the following mechanism underlying the immune microenvironment changes in SCI-IDS: the downregulation of peripheral CCR7 after acute SCI leads to the downregulation of Tfh cells through chemokine signaling pathway, causing the development of SCI-IDS and aggravating acute SCI.

Currently, limited and diverse studies are present on the immune microenvironment in SCI-IDS. Exploring the pathogenesis and therapeutic direction of SCI-IDS and systematically interpreting the theory of the immune microenvironment can fill the gap of SCI-IDS in terms of targeted therapy and elaboration of multi-omics pathological mechanisms. We hoped our study to pave the way to precise diagnosis and targeted treatment of acute SCI to ultimately reduce the mortality rate of acute SCI patients, and to provide a theoretical basis for future studies.

The present study had some limitations. First, the research was based on bioinformatics analysis, and the findings must be validated in animal and human experiments and in clinical practice. Second, the number of samples included in each group was small. Third, although the clinical prediction model had a high degree of agreement (area under the ROC curve of 0.892), its detection power must be improved by combining data from different studies. Fourth, because of the heterogeneity of acute SCI and inadequate clinical data, not all acute SCI patients experience significant systemic immunosuppression, and additional immunosuppressive features of acute SCI patients should be examined in future subgroup analyses.

## Conclusion

In conclusion, CCR7 was determined as a potential biomarker of acute SCI. CCR7 counteracts SCI-IDS by activating chemokine signaling in Tfh cells. We hope our finding will pave the way to an effective treatment for patients with acute SCI. The specific pathogenesis and molecular targets for SCI-IDS remain to be validated.

## Data availability statement

The original contributions presented in this study are included in the article/Supplementary material, further inquiries can be directed to the corresponding author.

## Author contributions

CL and CW helped in the conception and design, data acquisition, analysis and interpretation, and critical revision of the article and final approval. GX, YL, JC, JZ, HH, and CJ helped in the data acquisition and final approval. ZC helped in the data acquisition, analysis, and drafting and critical revision of the article and obtained final approval. All authors have approved the submitted version of the manuscript.

## Footnotes

1
https://www.ncbi.nlm.nih.gov/geo/

## Supplementary material

Supplementary Figure 1 The intersection of differentially expressed genes after acute SCI. **(A)** A venn diagram of differentially expressed genes in the HC, TC, and SCI groups. **(B)** A venn diagram of up-regulated differentially expressed genes in the HC, TC, and SCI groups. **(C)** A venn diagram of down-regulated differentially expressed genes in the HC, TC, and SCI groups.

Supplementary Figure 2 Functional enrichment analysis of differential genes. **(A–C)** Bubble diagram of the first 15 biological processes, molecular functions, and cellular components items, with horizontal coordinates indicating GeneRatio, vertical coordinates indicating gene ontology (GO) terms, dot size indicating the number of genes, and dot color indicating adj *p*-value. **(D)** Circle diagram of the first eight biological processes items, with the outer circle dot color representing upregulated and downregulated genes and the inner circle color representing activation or repression of GO terms. **(E)** Bubble diagram of the first 15 KEGG pathways, with horizontal coordinates indicating GeneRatio, vertical coordinates indicating KEGG pathways, dot size indicating the number of genes, and dot color indicating adj *p*-value. **(F)** String diagram of the first eight KEGG pathways, the left outer half-circle represents genes within the pathways, the color indicates log fold changes (logFC), the right outer half-circle color indicates KEGG pathways, and the inner connecting line indicates the association of KEGG pathways with genes.

Supplementary Figure 3 Differential expression and correlation analysis of CCR7. **(A)** Scatter plot of correlation analysis between CCR7 and ASIA classification. *R* denotes the correlation coefficient, and *P* denotes the *p*-value. Despite the fact that *P* < 0.05 is considered significantly correlated, *P* = 0.053 is considered statistical error interference. Therefore, CCR7 and ASIA scores were still considered significantly correlated. **(B)** Differential expression of CCR7 in the ASIA-high and ASIA-low groups. The number above the box indicates the *p*-value.
