# Supplementary material for: CCR7-mediated T follicular helper cell differentiation is associated with the pathogenesis and immune microenvironment of spinal cord injury-induced immune deficiency syndrome
Source: Front Neurosci. 2022 Oct 14;16:1019406. doi: 10.3389/fnins.2022.1019406 (PMC9615471; doi:10.3389/fnins.2022.1019406)
Supplement: Supplementary Figure 1 — The intersection of differentially expressed genes after acute SCI. (A) A venn diagram of differentially expressed genes in the HC, TC, and SCI groups. (B) A venn diagram of up-regulated differentially expressed genes in the HC, TC, and SCI groups. (C) A venn diagram of down-regulated differentially expressed genes in the HC, TC, and SCI groups. [file Data_Sheet_1.docx]

Supplementary Material


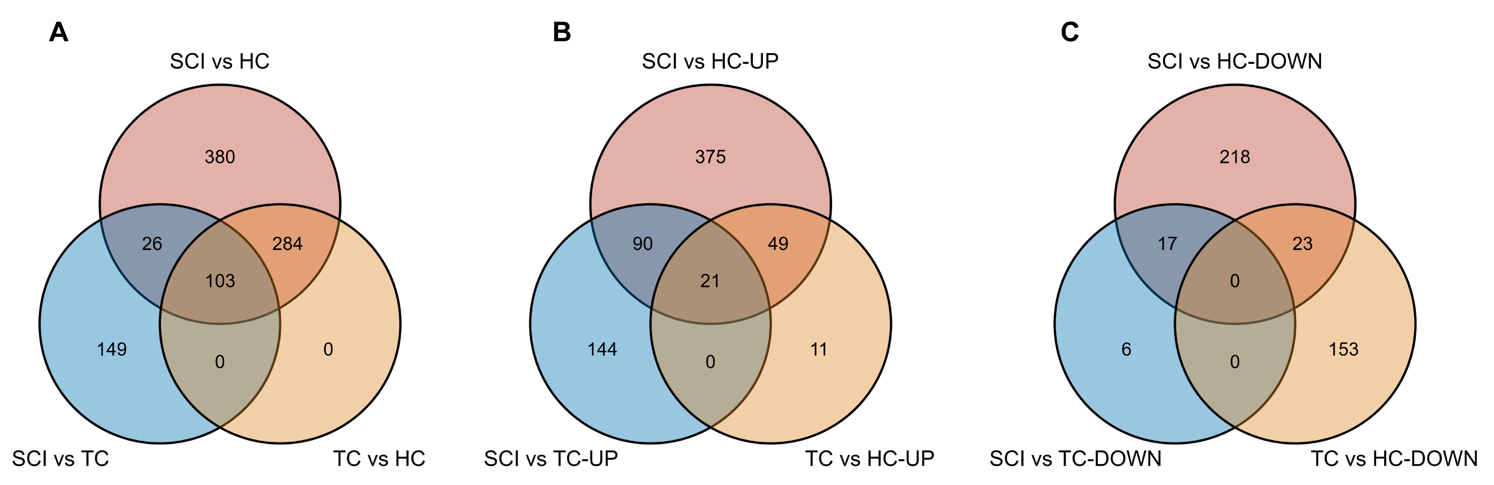


**Supplementary Figure 1.** The intersection of differentially expressed genes after acute SCI. **(A)** A venn diagram of differentially expressed genes in the HC, TC, and SCI groups. **(B)** A venn diagram of up-regulated differentially expressed genes in the HC, TC, and SCI groups. **(C)** A venn diagram of down-regulated differentially expressed genes in the HC, TC, and SCI groups.


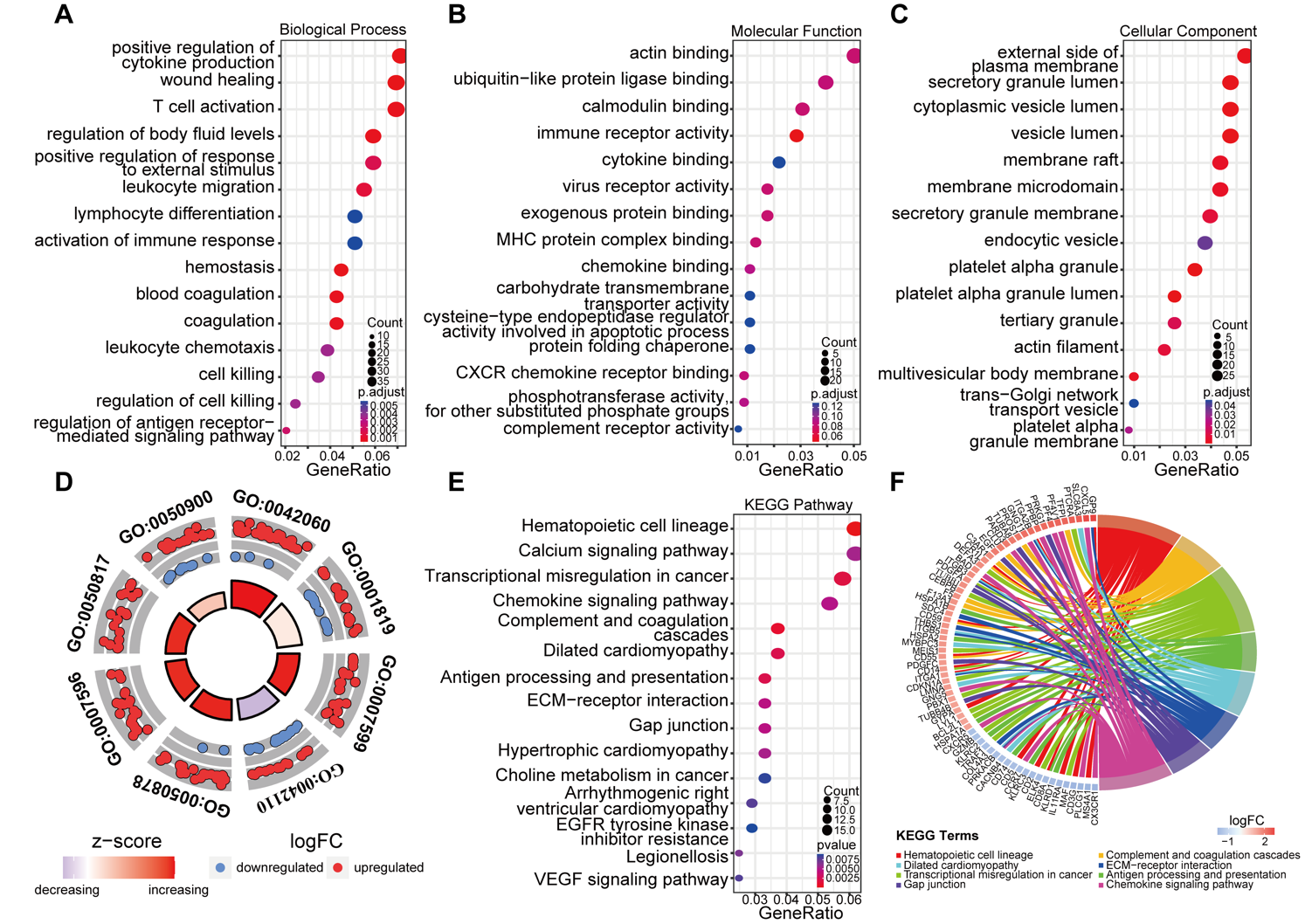


**Supplementary Figure 2.** Functional enrichment analysis of differential genes. **(A-C)** Bubble diagram of the first 15 biological processes, molecular functions, and cellular components items, with horizontal coordinates indicating GeneRatio, vertical coordinates indicating gene ontology (GO) terms, dot size indicating the number of genes, and dot color indicating adj *p*-value. **(D)** Circle diagram of the first eight biological processes items, with the outer circle dot color representing upregulated and downregulated genes and the inner circle color representing activation or repression of GO terms. **(E)** Bubble diagram of the first 15 KEGG pathways, with horizontal coordinates indicating GeneRatio, vertical coordinates indicating KEGG pathways, dot size indicating the number of genes, and dot color indicating adj *p*-value. **(F)** String diagram of the first eight KEGG pathways, the left outer half-circle represents genes within the pathways, the color indicates log fold changes (logFC), the right outer half-circle color indicates KEGG pathways, and the inner connecting line indicates the association of KEGG pathways with genes.


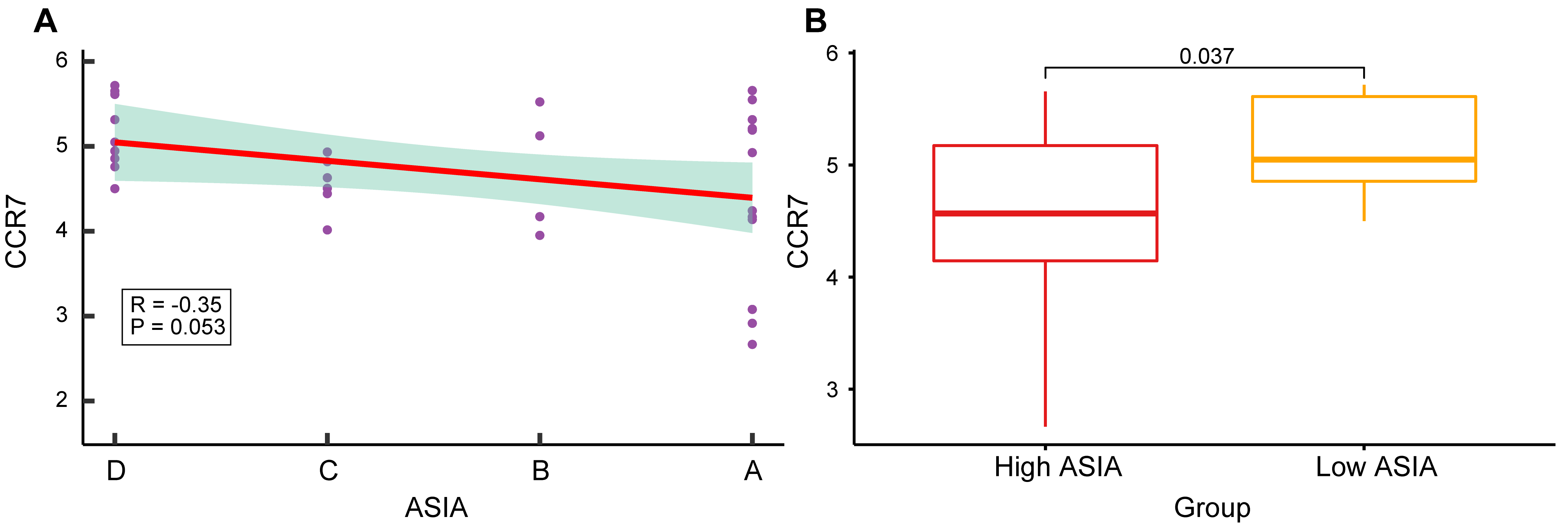


**Supplementary Figure 3.** Differential expression and correlation analysis of CCR7. **(A)** Scatter plot of correlation analysis between CCR7 and ASIA classification. R denotes the correlation coefficient, and P denotes the *p*-value. Despite the fact that P < 0.05 is considered significantly correlated, P = 0.053 is considered statistical error interference. Therefore, CCR7 and ASIA scores were still considered significantly correlated. **(B)** Differential expression of CCR7 in the ASIA-high and ASIA-low groups. The number above the box indicates the *p*-value.

**Supplementary Table 1.** Genes with significant and specific changes in expression after SCI

| **Gene Symbol** | | | | |
| --- | --- | --- | --- | --- |
| GNG5 | BAD | PLCG1 | CX3CR1 | PRKACB |
| CCR7 | CXCR5 | PARD3 | PF4 | CXCL5 |
| GNG11 | PF4V1 | PPBP | ANO10 | TLR8 |
| PLD1 | DYNLT1 | TXN | EXOC6 | CLTCL1 |
| PRDM11 | STK3 | DNASE1L1 | HSPA1B | GPAT3 |
| CD55 | MAD2L2 | NLRC3 | HMGB2 | FPR1 |
| GCA | LTB4R | RNF125 | MCTP2 | CD63 |
| CARD6 | AAK1 | TRIB2 | ARHGAP24 | CEBPB |
| ELK4 | SRPK1 | PPP1R3D | MPRIP | DRAM1 |
| ETS2 | BASP1 | SERPINB1 | MDN1 | NDUFB3 |
| TRAF5 | ANKH | SLC11A1 | CDKN2D | TMCO3 |
| LY96 | SMARCD3 | GNA15 | TNFSF13 | ANKRD36 |
| CBLB | NFE2 | LINC00342 | TNIK | MSRB1 |
| TRIQK | XRCC4 | MAF | CHPT1 | MTX1 |
| PRKCH | RFTN1 | SIDT1 | BCL11A | AUTS2 |
| RNF144A | F5 | CBX5 | PGS1 | FLVCR2 |
| GVINP1 | LOC645513 | SPATA41 | CD14 | SLC9A7P1 |
| ZFP36L2 | B9D2 | JMY | ST6GAL1 | AGFG1 |
| TSEN34 | GBGT1 | KLHL3 | AP3S2 | MANSC1 |
| FAM151B | BATF | VNN2 | SLC22A15 | AFF2 |
| SFXN5 | RNF43 | SGMS2 | PYCARD | IER3 |
| PECR | ANKS6 | DOCK9 | LOC728743 | CD3G |
| NLRP12 | HSPA1A | WDR13 | NOG | DGAT2 |
| KCNA3 | ZMAT5 | GPR160 | TMEM260 | ANTXRLP1 |
| ZNF827 | FPR2 | MILR1 | TRAF1 | IL11RA |
| MXD3 | NCF4 | PWARSN | CLEC2D | SLC38A1 |
| CCDC71L | KBTBD7 | SMAD3 | GBA | CCDC88C |
| OAT | GAS7 | OTUB2 | SORT1 | AGTRAP |
| ANKRD36C | PAQR4 | SLC16A3 | LOC101927851 | HEG1 |
| FMNL3 | MKNK1 | TWF2 | TMEM91 | NQO2 |
| KLRD1 | ZXDB | FGGY | LRBA | PPP2R3B |
| CD59 | B4GALT5 | DPY19L3 | RIC3 | PLAC4 |
| LY9 | ALOX5AP | LOC100507642 | FAM160A2 | TMEM144 |
| ADAMTS10 | PNPLA1 | PRR5L | RNF175 | TPI1P2 |
| OPRL1 | CD82 | FBXO32 | GALNT3 | HRH2 |
| SLAMF6 | SCARNA20 | CLEC4E | DOK4 | ERLIN1 |
| SEC14L1P1 | SLFN12L | FSTL3 | AURKAIP1 | JOSD2 |
| PTGDR | TNFRSF25 | TBC1D4 | CD247 | MBOAT2 |
| CAMK2D | GOLGA7B | TBC1D8 | CARD11 | TUBB4B |
| ACVR1B | MAFG | ITPKC | MYO7B | UBASH3A |
| PCED1B | S1PR4 | PIM3 | MIR646HG | ATP8B2 |
| LBH | CAMK4 | SLC41A1 | DOK3 | PPP1R16B |
| LILRA6 | FCRL3 | CD5 | SKA3 | SLC5A9 |
| CYBA | EXT1 | MAGIX | TTC8 | AHNAK |
| RAB43 | B3GNT8 | KIF3C | GBAP1 | RASA4 |
| OSCAR | DOCK10 | CDC34 | MYBPC3 | NEDD4 |
| EPHX2 | DPM3 | EFNA1 | LRP3 | TNNI2 |
| STAT4 | LOC101928977 | ARID5A | ITGA1 | FBN2 |
| GAS6 | RPL3 | SPHK1 | LSMEM1 | PTPN4 |
| CDS1 | NOXRED1 | ST14 | SBNO2 | ABHD12B |
| TIMP1 | PHLDB2 | SBK1 | CD6 | LINGO3 |
| MCOLN2 | KREMEN1 | FLOT2 | DLGAP5 | OPTN |
| SLAMF1 | CCDC126 | NRADDP | CEP78 | AMOT |
| SYCP2 | NCALD | LYL1 | CEACAM6 | NT5DC2 |
| TIGIT | CDKN3 | CTSD | KLRG1 | DEPDC1B |
| DLG3 | NR1D2 | PLP2 | FAM20C | TPD52 |
| CD2 | TC2N | FMN1 | ZNF775 | RFX2 |
| SH2D1A | KIF21A | BNC2 | NAPRT | SH2D2A |
| TOX2 | DNAH6 | ABCB1 | LOC101928075 | SRGAP3 |
| CMTM1 | CNTNAP3 | INPP4B | THEMIS | DPP4 |
| CATIP | C11orf42 | SNORA67 | ANK3 | PRUNE2 |
| NR1I3 | TNFRSF12A | GRAMD1A | MYO7A | ARL10 |
| TBKBP1 | GCSAM | LOC100288846 | CNTNAP3B | HSD3B7 |
| GBP5 | C3AR1 | SNORA80A | SNORA15 | TMEM150B |
| MIR7848 | TRIM9 | SAMD12 | COLGALT2 | SYTL2 |
| OSBPL10 | LINC01127 | KY | LIMS2 | SNORA71A |
| ENTPD7 | CNR2 | ZNF860 | S1PR5 | VPS9D1 |
| ABCD2 | SCARNA18 | FSIP2 | NSG1 | PTGS2 |
| GCNT4 | OBSCN | CD160 | PTPRK | CARNS1 |
| TREML3P | SNORA21 | GZMB | SIGLEC8 | UPB1 |
| CD74 | GK3P | CACNA1I | SLED1 | SCARNA23 |
| NAP1L2 | KLRF1 | COL4A3 | RAP1GAP | RGS9 |
| NDFIP2 | AATK | CEACAM1 | SNORA70 | CAND1.11 |
| SCART1 | CD8A | KLRB1 | MS4A1 | ACVR1C |
| SNORA2A | PPP2R2B | GRINA | SNORA5A | COL19A1 |
| CR1L | SMTNL1 | SCARNA11 | SCARNA8 | SNHG19 |
| RNU4ATAC | SNORA71B | SNORA24 | SCARNA16 | ZNF467 |
| ENPP5 | TP53I11 | TMTC1 | RNU11 | SNORA10 |
| SLC2A14 | SNORD74 | LCN2 | RNU6ATAC | SNORA71D |
| SNORA64 | MAP3K7CL | RNY5 | SNORA16A | PRR12 |
| LINC00664 | KLRC3 | GRAMD1C | GZMK | MIR3609 |
| FCER1A | SCARNA2 | ANKRD34B | KLRC2 | RNY4 |
| GZMH | DEFA3 | P2RY1 | PDGFA | LTBP1 |
| ABLIM3 | TFPI | TREML1 | SDC4 | STOM |
| SLC8A3 | NT5M | TSPAN9 | LINC00989 | TMEM40 |
| FKBP1B | CTTN | MED12L | P2RY12 | GP9 |
| PLOD2 | LY6G6F | CMTM5 | MMRN1 | TDRP |
| PTCRA | PDLIM1 | SMOX | ESAM | CLU |
| GNAZ | WASF3 | EGF | PLCH1 | PDE3A |
| LINC01359 | PCSK6 | COL24A1 | MGLL | ENKUR |
| PROS1 | DTL | ARHGAP6 | MEIS1 | CLDN5 |
| AQP10 | CDKN1A | TRAPPC3L | TUBA8 | MIR3916 |
| THBS1 | ASAP2 | PKHD1L1 | DENND2C | CABP5 |
| SLFN14 | PRKG1 | KIF15 | CTDSPL | MYCT1 |
| MYL9 | HRAT92 | NCAPG | TSPAN33 | FSTL1 |
| ITGB5 | LOC101927854 | NAT8B | RHOBTB1 | MYLK |
| RAB27B | CALD1 | TNFSF4 | ITGA2B | NCR1 |
| EGFL7 | RPGRIP1 | SPARC | SCARNA1 | SH3BGRL2 |
| CXCR2P1 | DNM3 | PRTFDC1 | CHGA | GAS2L1 |
| PDGFC | RGS6 | EPHB1 | BEND2 | CCNB1 |
| TUBB1 | ITGB3 | POLE2 | JAM3 | MORC1 |
| PBX1 | RNU12 | BCL2L1 | VIL1 | GATA1 |
| CPNE5 | F13A1 | SNORD55 | VEPH1 | MMD |
| HGD | PRKAR2B | LMNA | SNORA40 | LAPTM4B |
| SNORA7B | NCKAP1 | TMCC2 | RANBP17 | CACNB4 |
| ARMC3 | HEMGN | NEAT1 | SNORA65 | SNORA60 |
| VN1R1 | ABCC3 | SNORA80E | SNORD22 | SNORD15B |
| SNORA73B | EPB42 | SNORA44 | CCDC175 | SNORA66 |
| SNORA26 | PDZK1IP1 | PLA2G4C | NRGN | SNORA9 |
| SNORD67 | C15orf54 | SNORA11 | CA1 | SELENBP1 |
| SNORA74B | DNAJC6 | HSPA2 | ALAS2 | GYPA |

**Supplementary Data 1.** Fifteen MCODE modules in the PPI network.

**Supplementary Data 2.** Genes (n = 5094) that were highly correlated with ASIA scores base on univariate logistic regression analysis.
